# Women’s attitudes towards negotiating safe sexual practices in Nigeria: Do family structure and decision-making autonomy play a role?

**DOI:** 10.1186/s12905-022-01602-7

**Published:** 2022-01-22

**Authors:** Chukwuechefulam Kingsley Imo, Clifford O. Odimegwu, Nicole De Wet-Billings

**Affiliations:** 1 Department of Sociology, Faculty of the Social Sciences, Adekunle Ajasin University, P. M. B. 001, Akoko-Akungba, Ondo State Nigeria; 2 Demography and Population Studies Programme, Schools of Public Health and Social Sciences, University of the Witwatersrand, Johannesburg, South Africa

**Keywords:** Family structure, Decision-making, Attitudes, Safe, Sexual practices, Nigeria

## Abstract

**Background:**

The risk of contracting sexually transmitted infections (STIs), including human immunodeficiency virus (HIV) is related to women’s sexual attitudes, beliefs, and power dynamics within marriages in developing countries. Despite the interventions towards improving women’s sexual health and well-being, women are disproportionately affected by the risk of STIs transmission compared with their male counterparts in most sub-Saharan African countries including Nigeria. This study examined the roles of family structure and decision-making autonomy on women’s attitudes towards negotiating safe sexual practices in Nigeria.

**Methods:**

The study involved analyses of data from a nationally representative and weighted sample size of 28,219 ever-married/cohabiting women aged 15–49 years from the 2018 Nigeria Demographic and Health Survey. Descriptive and statistical analyses were carried out, including frequency tables, Pearson’s chi-square test, and multivariable binary logistic regression model.

**Results:**

The overall prevalence of having positive attitudes towards negotiating safe sexual practices were 76.7% and 69.6% for a wife justified in asking the husband to use a condom if he has an STI and refusing to have sex with the husband if he had sex with other women, respectively. The results further showed that polygamous unions negatively influenced urban and rural women’s attitudes towards negotiating safe sexual practices, but women’s decision-making autonomy on how to spend their earnings was found to be a protective factor for having positive attitudes towards negotiating safe sexual practices with partners. Surprisingly, there were significant variations in attitudes towards negotiating safe sexual practices among urban and rural women who enjoyed decision-making autonomy on their healthcare (aOR 1.70; CI 1.32–2.18 and aOR 0.52; CI 0.44–0.62, respectively). Plausibly, such women might have constrained them to compromise their sexual relationships for fear of being neglected by partners.

**Conclusion:**

The outcomes of this study have some policy implications for both maternal and child health. There is the need to intensify programmes aimed at improving women’s sexual health and rights towards achieving sustainable development goals of preventing deaths of newborns, ending STIs and creating gender in Nigeria.

## Background

In the context of global health priorities, sexually transmitted infections (STIs), including human immunodeficiency virus (HIV) remain reproductive and public health concerns. The STIs, particularly HIV, disproportionately affect women, compared to their male counterparts [1], and the risk of contracting the infections is related to sexual attitudes, beliefs, and power dynamics that exist among sex partners [2]. As a consequence, the decision-making power of a woman regarding safe sexual practice influences her competence in controlling the health outcomes, especially the chances of contracting STIs and experiencing unintended pregnancies in marriage [3].

Marriage is a social union that influences spousal communication on sexual and reproductive health activities. Hence, denying partner sex is discouraged, while unprotected sexual intercourse is perceived in many cultures as strengthening the marriage institution [4]. An emerging study has shown that women’s power and autonomy is favourably related to better sexual and reproductive health, including the use of contraceptives [5]. However, the patriarchal society present in many sub-Saharan African countries, encourage competition among women to win husbands’ love and women may find it difficult to oppose men’s positions regarding sexual matters [6]. Women in polygamous unions are less likely to use contraceptive methods and safer sex dialogue is regarded as appropriate only between the husband and younger wife because she is perceived at risk of engaging in extramarital relations [7].

Cultural and institutional norms like male-headed households and gender-based power inequalities within sexual relationships negatively affect women’s sexual and reproductive health [8], as well as explain the reason for engaging in unsafe sexual activities in marriage [9]. Most African women in marital or sexual unions abide by their partners’ wishes and compromise their positions on reproductive issues to ensure stability in relationships [10], because of fear of being neglected by one’s partner and losing him to other women [11]. The unequal power over sex is also displayed by men’s unwillingness to practice safe sex and demands in that regard by women are often portrayed as a sign of mistrust or infidelity [12]. Thus, women are disproportionately exposed to STIs, particularly HIV compared with their male counterparts [13].

In view of placing a priority on health, there have been numerous plans by countries including Nigeria to implement international resolution through policy formulations and implementation to make forcible sexual intercourse against women socially and culturally unacceptable to minimise STIs. Despite previous studies on women’s decision-making power and safer sex negotiation in Nigeria [8, 14, 15], the comparison and measurement of the influence of family structure and decision-making autonomy on women’s attitudes towards negotiating safe sexual practices have been quite limited. This is evident in Nigeria where women are disproportionally affected by HIV accounting for about 56% of adults living with HIV, as well as the percentage of HIV-exposed infants tested for HIV before eight weeks of age standing at 18% in 2018 [1]. Consequently, one of the major factors that contribute to the spread of STIs in a developing country like Nigeria is the inability to negotiate safe sexual practices among women.

Notwithstanding that 69.7% and 77.3% of married women believe that it would be justified for a woman to ask the husband to use condoms if he has STI and in refusing to have sexual intercourse with her husband if she knows that he has sexual intercourse with other women, respectively, modern contraceptive use is 12% and 17% for any method [16]. This might account for about 56% of adults living with HIV and the percentage of HIV-exposed infants tested for HIV before eight weeks of age standing at 18% in 2018 in Nigeria [1]. Therefore, the women-based approach with emphasis on the theory of reasoned action establishing that beliefs, attitudes, intentions and behaviours form a causal chain [17], provides a novel opportunity to understand the processes related to unsafe sexual relationships in most marriages. This study, therefore, examined the roles of family structure and decision-making autonomy on women’s attitudes towards negotiating safe sexual practices in Nigeria. The outcome is essential to the design and assessment of interventions to improve sexual health, as well as achieve sustainable development goals (SDGs) target of ending STIs including HIV and reducing the burden of babies born with infections from STIs in Nigeria.

## Methods

### Data source and design

The data for this study were obtained from the individual recode data file of the 2018 Nigeria Demographic and Health Survey (NDHS). The NDHS 2018 is a cross-sectional study and the survey provides up-to-date information on demographic and health indicators in Nigeria [16]. A representative sample of 41,668 households was selected for the survey and the data were generated from 41,821 women aged 15–49 and 13,311 men aged 15–59. A detailed report of the methods and procedures of data assemblage for 2018 NDHS has been documented elsewhere [16]. The analyses for this study covered a weighted sample of 28,219 women (urban—10,171 and rural—18,048) who reported being married or living with their partners within the 5 years preceding the 2018 survey (i.e. 2013–2018).

### Variables measurements

#### Outcome variables

The outcome variables were two basic attitudinal questions on women’s negotiation for safe sexual practices: These questions are (i) wife justified asking that they use a condom if she knows that her husband has an STI and (ii) wife justified refusing to have sex with the husband if he had sex with other women which have two responses of ‘yes’ and ‘no’. The respondents whose responses were in affirmative were classified as having ‘positive’ attitudes towards negotiating safe sexual practices—coded as 1, otherwise classified as having ‘negative’—coded as 0.

#### Explanatory variables

The main explanatory variables were ‘family structure’ and ‘decision-making autonomy’. In this context, family structure represents monogamous or polygamous union which was derived from the question—number of other wives [18]. The respondents who reported having no other wives were categorised as being in ‘monogamous unions’ and those with other wives as ‘polygamous unions’. Women decision-making autonomy depicts as the extent of independence on finances, matters pertaining to her health and that of the households without having to obtain permission from partners [19], was derived from the following three subjects: (1) the person who usually decides on respondent’s health care, (2) the person who usually decides on large household purchases, and (3) the person who usually decides how to spend respondent’s earnings. The possible answers were regrouped as respondent alone, respondent and husband/partner jointly and husband/partner alone/someone else. Therefore, respondents who reported making independent decisions (alone) on the decision-making subjects represent ‘decision-making autonomy’.

The co-variables included in the analysis were age, marital status, educational attainment, employment status, wealth quintile and region. Some variables were regrouped to make interpretation simpler and meaningful. The documented significant association with sexual practices in the literature and their availability in the dataset guided the selection of all the variables.

#### Statistical analysis

For this study, the dataset was carefully checked for missing values that were excluded from the analyses and weighted with the appropriate sampling weights as per the Demographic and Health Survey (DHS) sampling scheme before the analyses using Stata software (version 14). The analyses were done at univariate, bivariate and multivariate levels. At the bivariate level, the Pearson chi-square test was adopted to investigate urban–rural disparity of attitudes towards negotiating safe sexual practices for all the explanatory variables. The multivariable binary logistic regression models were used at the multivariate level, to measure the odds ratio (OR) of the association between women’s attitudes towards negotiating safe sexual practices and the explanatory variables. The results were expressed as OR with 95% confidence intervals (CI) and an explanatory variable with OR greater than 1.00 implied an increased likelihood of the outcome (attitudes towards negotiating safe sexual practices), while it is the opposite when the OR is less than 1.00 [20].

## Results

### Distribution of the variables of the study population sample

Description of respondents by socio-economic and demographic characteristics is presented in Table 1 with weighted frequencies and percentages. Overall, the mean age of the women was 32 years, while urban and rural women were 33 and 31 years, respectively. There were slightly older rural women in the sample than their urban counterparts (43.9% vs. 37.2%). With respect to educational attainment, the largest proportion of women had no formal education, with a huge variation by place of residence. For instance, 56.7% of the rural women had no formal education relative to 61.8% of their urban counterparts with secondary/tertiary education. Overall, about 70% of the women reported being employed. The result was slightly higher among urban women (75.7%) compared with 67% of women residing in rural areas. The largest proportion of women was found in the lowest wealth quintile households, with the majority of urban women (67.1%) found in the highest wealth quintile households, compared to 61.0% of rural women in the lowest category. Considering region of residence, the proportions of the women ranged from 28.5% in the North-west and 10.0% in the South-south with variations across the place of residence.

**Table 1:** Percentage distribution of respondents’ socio-economic and demographic characteristics, NDHS 2018

Characteristics	Urban and rural	Urban	Rural
	N (%)	N (%)	N (%)
Age Mean	32 years	33 years	31 years
15–24	5,990 (21.2)	1468 (14.4)	4522 (25.1)
25–34	11,039 (39.1)	4234 (41.6)	6805 (37.7)
35 and above	11,190 (39.7)	4469 (43.9)	6721 (37.2)
Educational attainment			
No education	12,427 (44.0)	2196 (21.6)	10,231 (56.7)
Primary	4669 (16.6)	1686 (16.6)	2983 (16.5)
Secondary/tertiary	11,123 (39.4)	6289 (61.8)	4834 (26.8)
Employment status			
Not working	8425 (29.9)	2470 (24.3)	5955 (33.0)
Currently working	19,794 (70.1)	7701 (75.7)	12,093 (67.0)
Wealth quintile			
Lowest	12,348 (43.8)	1340 (13.2)	11,008 (61.0)
Middle	5714 (20.2)	1999 (19.7)	3715 (20.6)
Highest	10,157 (36.0)	6832 (67.1)	3325 (18.4)
Region			
North-central	5153 (18.3)	1604 (15.8)	3549 (19.7)
North-east	5492 (19.5)	1121 (11.0)	4371 (24.2)
North-west	8049 (28.5)	1977 (19.4)	6072 (33.6)
South-east	3141 (11.1)	1994 (19.6)	1147 (6.4)
South-south	2813 (10.0)	948 (9.3)	1865 (10.3)
South-west	3571 (12.6)	2527 (24.9)	1044 (5.8)

### Family structure, decision-making measures and negotiating safe sexual practices

Table 2 presents the distribution results of respondents by family structure, decision-making measures and attitudes towards negotiating safe sexual practices. The results indicated that 69.9% of the women were found in monogamous unions, with slightly more rural women than their urban counterparts in polygamous unions (34.8% and 21.7%, respectively). For decision-making measures, the largest proportion of the decisions on women’s healthcare was made by their husbands (56.0%). A similar result was recorded in rural areas, while 43.5% of the decisions on women’s healthcare were jointly made with husbands. The results in Table 2 further showed that women lack decision-making autonomy on large household purchases, as such decisions were solely made by their husbands. On the other hand, the largest proportion of the women enjoyed decision-making autonomy on how to spend their earnings (69.5%). A similar result was observed among urban and rural women with a slight difference (67.7% and 70.9%, respectively). Concerning attitudes towards negotiating safe sexual practices, over two-thirds of the women believed that it would be justified for a woman to ask the husband to use condoms if he has STI and refuse to have sexual intercourse with her husband if she knows that he has sexual intercourse with other women.

**Table 2:** Distribution of respondents’ family structure, decision-making measures and negotiating safe sexual practices, NDHS 2018

Characteristics	Urban and rural	Urban	Rural
	N (%)	N (%)	N (%)
Family structure			
Monogamous	19,721 (69.9)	7959 (78.3)	11,762 (65.2)
Polygamous	8498 (30.1)	2212 (21.7)	6286 (34.8)
Decision on respondent's healthcare			
Husband/partner alone and other	15,810 (56.0)	4342 (42.6)	11,468 (63.5)
Jointly	9554 (33.9)	4419 (43.5)	5135 (28.5)
Alone	2855 (10.1)	1410 (13.9)	1445 (8.0)
Decision on large household purchase			
Husband/partner alone and other	16.681 (59.2)	4809 (47.3)	11,872 (65.8)
Jointly	9889 (35.0)	4555 (44.8)	5334 (29.6)
Alone	1649 (5.8)	807 (7.9)	842 (4.7)
Decision on how to spend respondent's earning			
Husband/partner alone and other	1651 (9.56)	493 (6.7)	1158 (11.6)
Jointly	3610 (20.9)	1865 (25.6)	1745 (17.5)
Alone	12,009 (69.5)	4940 (67.7)	7069 (70.9)
Wife justified asking the husband to use a condom if he has STI			
No	6575 (23.3)	1832 (18.0)	4743 (26.3)
Yes	21,644 (76.7)	8339 (82.0)	13,305 (73.7)
Refusing to have sex with the husband if he has sex with other women			
No	8575 (30.4)	2871 (28.2)	5704 (31.6)
Yes	19,644 (69.6)	7300 (71.8)	12,344 (68.4)

### Bivariate association of women’s attitudes towards negotiating safe sexual practices with all the explanatory variables

Table 3 presents the unadjusted logistic regression results showing the association between women’s attitudes towards negotiating safe sexual practices and all the explanatory variables.

**Table 3:** Unadjusted logistic regression analysis of the association between attitudes towards negotiating safe sexual practices and explanatory variables, NDHS 2018

Characteristics	Wife justified asking the husband to use a condom if he has STI		Refusing to have sex with husband if he has sex with other women	
	Urban	Rural	Urban	Rural
	OR(95% CI)	OR(95% CI)	OR(95% CI)	OR(95% CI)
Family structure				
Monogamous (RC)	1.00	1.00	1.00	1.00
Polygamous	0.71 (0.63–0.80)***	0.82 (0.76–0.87)***	0.73 (0.66–0.81)***	0.86 (0.80–0.91)***
Decision on respondent's healthcare				
Husband/partner alone and other (RC)	1.00	1.00	1.00	1.00
Jointly	1.53 (1.38–1.71)***	1.35 (1.25–1.45)***	0.98 (0.89–1.07)	1.09 (1.02–1.18)*
Alone	2.31 (1.93–2.77)***	1.48 (1.29–1.68)***	0.93 (0.82–1.07)	0.63 (0.56–0.71)***
Decision on large household Purchase				
Husband/partner alone and other (RC)	1.00	1.00	1.00	1.00
Jointly	1.41 (1.27–1.57)***	1.36 (1.26–1.47)***	0.97 (0.89–1.06)	1.15 (1.07–1.23)***
Alone	1.67 (1.35–2.07)***	1.68 (1.41–2.00)***	0.93 (0.79–1.09)	0.76 (0.66–0.88)***
Decision on how to spend respondent's earning				
Husband/partner alone and other (RC)	1.00	1.00	1.00	1.00
Jointly	2.45 (1.96–3.07)***	3.61 (3.07–4.24)***	1.40 (1.13–1.72)**	2.53 (2.17–2.95)***
Alone	2.75 (2.24–3.38)***	3.59 (3.16–4.08)***	1.43 (1.18–1.74)***	2.45 (2.16–2.78)***
Age				
15–24 (RC)	1.00	1.00	1.00	1.00
25–34	1.15 (0.99–1.34)	0.99 (0.91–1.08)	0.95 (0.83–1.09)	0.84 (0.77–0.91)***
35 and above	1.13 (0.97–1.31)	1.00 (0.92–1.09)	0.95 (0.84–1.09)	0.84 (0.78–0.92)***
Education attainment				
No education (RC)	1.00	1.00	1.00	1.00
Primary	1.60 (1.38–1.86)***	1.47 (1.33–1.61)***	1.21 (1.06–1.39)**	1.14 (1.04–1.24)**
Secondary/tertiary	2.83 (2.52–3.19)***	2.05 (1.88–2.23)***	1.51 (1.36–1.68)***	1.20 (1.12–1.30)***
Employment status				
Not working (RC)	1.00	1.00	1.00	1.00
Currently working	1.46 (1.30–1.63)***	1.29 (1.20–1.38)***	1.05 (0.95–1.16)	0.97 (0.91–1.04)
Wealth Quintile				
Lowest (RC)	1.00	1.00	1.00	1.00
Middle	1.30 (1.10–1.53)**	1.34 (1.23–1.46)***	1.31 (1.12–1.52)**	0.94 (0.87–1.02)
Highest	1.94 (1.69–2.23)***	1.71 (1.56–1.88)***	1.21 (1.07–1.37)***	1.09 (1.00–1.18)*
Region				
North-central (RC)	1.00	1.00	1.00	1.00
North-east	0.98 (0.82–1.18)	1.20 (1.09–1.32)***	1.88 (1.59–2.23)***	2.17 (1.98–2.38)***
North-west	1.13 (0.95–1.32)	1.13 (1.03–1.24)**	2.44 (2.10–2.83)***	2.28 (2.09–2.49)***
South-east	1.91 (1.59–2.28)***	2.99 (2.47–3.61)***	1.58 (1.38–1.82)***	2.35 (2.03–2.73)***
South-south	1.34 (1.09–1.65)**	1.10 (0.97–1.25)	1.45 (1.22–1.72)***	1.38 (1.23–1.54)***
South-west	1.25 (1.06–1.46)**	1.05 (0.90–1.22)	1.52 (1.33–1.73)***	2.04 (1.76–2.37)***

RC,  reference category

^*^p < 0.05; **p < 0.01; ***p < 0.001

#### Wife justified asking the husband to use a condom if he has STI

The results in Table 3 showed a significant influence of family structure and decision-making measures on believing that it would be justified for a woman to ask the husband to use condoms if he has STI. For instance, coming from polygamous unions was significantly found not to be a protective factor for believing that a wife is justified asking the husband to use a condom if he has STI among urban and rural women (OR 0.71; CI 0.63–0.80 and OR 0.82; CI 0.76–0.87, respectively). Our results further revealed that the likelihood of believing that it would be justified for a woman to ask the husband to use condoms if he has STI significantly increased among urban and rural women who enjoyed decision-making autonomy on their healthcare (OR 2.31; CI 1.93–2.77 and OR 1.48; CI 1.29–1.68, respectively). Similar results were observed among urban and rural women who made independent decisions on large household purchases and how to spend their earnings. The results in Table 3 further showed that except for age, all the co-variables were significantly associated with women believing that it would be justified for a woman to ask the husband to use condoms if he has STI.

#### Refusing to have sex with the husband if he had sex with other women

The results indicated that family structure and decision-making measures significantly influenced women’s attitudes towards negotiating for safe sexual practices in Table 3. For instance, our results showed that the likelihood of believing that a woman is justified in refusing to have sexual intercourse with her husband if she knows that he has sexual intercourse with other women significantly reduced among urban and rural women found in polygamous unions (OR 0.73; CI 0.66–0.81 and OR 0.86; CI 0.80–0.91, respectively). Similar significant results were observed among rural women who made independent decisions on their healthcare and large household purchases. On the other hand, the likelihood of believing that a woman is justified in refusing to have sexual intercourse with her husband if she knows that he has sexual intercourse with other women significantly increased among urban and rural women who made independent decisions on how to spend their earnings (OR 1.43; CI 1.18–1.74 and OR 2.45; CI 2.16–2.78, respectively). The results in Table 3 further showed that the women’s age, educational attainment, wealth quintile and region of residence were significantly associated with believing that a woman is justified in refusing to have sexual intercourse with her husband if she knows that he has sexual intercourse with other women.

### Multivariate association of women’s attitudes towards negotiating safe sexual practices with family structure and decision-making measures

Table 4 presents the adjusted results of the influence of family structure and women’s decision-making measures on attitudes towards negotiating safe sexual practices.

**Table 4:** Adjusted multivariable analyses of attitudes towards negotiating safe sexual practices and family structure and decision-making characteristics of respondents

Characteristics	Wife justified asking the husband to use a condom if he has STI		Refusing to have sex with husband if he has sex with other women	
	Urban	Rural	Urban	Rural
	aOR (95% CI)	aOR (95% CI)	aOR (95% CI)	aOR (95% CI)
Family structure				
Monogamous (RC)	1.00	1.00	1.00	1.00
Polygamous	0.71 (0.61–0.83)***	0.74 (0.67–0.81)***	0.65 (0.57–0.74)***	0.75 (0.68–0.82)***
Decision on respondent's healthcare				
Husband/partner alone and other (RC)	1.00	1.00	1.00	1.00
Jointly	1.23 (1.01–1.51)*	0.95 (0.81–1.10)	0.91 (0.77–1.08)	0.70 (0.61–0.80)***
Alone	1.67 (1.32–2.13)***	1.24 (1.03–1.50)*	0.81 (0.67–0.93)*	0.47 (0.40–0.55)***
Decision on large household purchase				
Husband/partner alone and other (RC)	1.00	1.00	1.00	1.00
Jointly	0.91 (0.74–1.10)	1.04 (0.89–1.21)	0.79 (0.67–0.93)**	1.18 (1.03–1.35)*
Alone	0.97 (0.74–1.29)	1.00 (0.78–1.27)	0.91 (0.74–1.13)	1.20 (0.97–1.49)
Decision on how to spend respondent's earning				
Husband/partner alone and other (RC)	1.00	1.00	1.00	1.00
Jointly	2.25 (1.76–2.87)***	3.47 (3.90–4.15)***	1.62 (1.29–2.03)***	2.79 (2.35–3.32)***
Alone	2.64 (2.14–3.25)***	3.63 (3.18–4.14)***	1.63 (1.33–1.99)***	2.79 (2.45–3.18)***

RC, reference category

*p < 0.05; ***p < 0.001

#### Wife justified asking the husband to use a condom if he has STI

The results in Table 4 showed that urban and rural women found in polygamous unions were less likely to believe that it would be justified for a woman to ask the husband to use condoms if he has STI (aOR 0.71; CI 0.61–0.83 and aOR 0.74; CI 0.67–0.81, respectively). Also, the likelihood of believing that it would be justified for a woman to ask the husband to use condoms if he has STI significantly increased among urban and rural women who made independent decisions on their healthcare (aOR 1.67; CI 1.32–2.13 and aOR 1.24; CI 1.03–1.50, respectively) and how to spend their earnings (aOR 2.64; CI 2.14–3.25 and aOR 3.63; CI 3.18–4.14, respectively).

#### Refusing to have sex with the husband if he had sex with other women

The results in Table 4 further confirmed that the likelihood of believing that a woman is justified in refusing to have sexual intercourse with her husband if she knows that he has sexual intercourse with other women significantly reduced among urban and rural women found in polygamous unions (aOR 0.65; CI 0.57–0.74 and aOR 0.75; CI 0.68–0.82, respectively). Surprisingly, similar results were observed among urban and rural women who made independent decisions on their healthcare (aOR 0.81; CI 0.67–0.93 and aOR 0.47; CI 0.40–0.55, respectively). But the likelihood of believing that a woman is justified in refusing to have sexual intercourse with her husband if she knows that he has sexual intercourse with other women significantly increased among urban and rural women who made independent decisions on their healthcare (aOR 1.63; CI 1.33–1.99 and aOR 2.79; CI 2.45–3.18, respectively).

### Multivariate association of women’s attitudes towards negotiating safe sexual practices with family structure, decision-making measures and selected co-variables

Table 5 presents the results of the adjusted association between women’s attitudes towards negotiating safe sexual practices and decision-making measures, as well as significant co-variables using multivariable analysis in Table 3.

**Table 5:** Adjusted multivariable analyses of attitudes towards negotiating safe sexual practices and explanatory variables and selected co-variables

Characteristics	Wife justified asking the husband to use a condom if he has STI		Refusing to have sex with husband if he has sex with other women	
	Urban	Rural	Urban	Rural
	aOR (95% CI)	aOR (95% CI)	aOR (95% CI)	aOR (95% CI)
Family structure				
Monogamous (RC)	1.00	1.00	1.00	1.00
Polygamous	0.89 (0.76–1.05)	0.81 (0.73–0.90)***	0.62 (0.54–0.71)***	0.73 (0.66–0.81)***
Decision on respondent's healthcare				
Husband/partner alone and other (RC)	1.00	1.00	1.00	1.00
Jointly	1.25 (1.01–1.55)*	0.90 (0.77–1.05)	0.99 (0.83–1.18)	0.72 (0.63–0.84)***
Alone	1.70 (1.32–2.18)***	1.21 (1.00–1.47)	0.91 (0.76–1.10)	0.52 (0.44–0.62)***
Decision on large household purchase				
Husband/partner alone and other (RC)	1.00	1.00	1.00	1.00
Jointly	0.82 (0.66–1.00)	0.98 (0.84–1.15)	0.82 (0.70–0.97)*	1.19 (1.03–1.37)*
Alone	0.89 (0.67–1.18)	0.92 (0.71–1.18)	0.99 (0.79–1.23)	1.29 (1.04–1.61)*
Decision on how to spend respondent's earning				
Husband/partner alone and other (RC)	1.00	1.00	1.00	1.00
Jointly	2.14 (1.67–2.75)***	3.03 (2.52–3.65)***	1.59 (1.27–2.00)***	2.60 (2.18–3.10)***
Alone	2.85 (2.29–3.56)***	3.64 (3.17–4.17)***	1.42 (1.15–1.74)**	2.51 (2.19–2.86)***
Age				
15–24 (RC)	–	–	1.00	1.00
25–34	–	–	0.97 (0.80–1.18)	0.88 (0.78–1.00)
35 and above	–	–	1.10 (0.91–1.33)	0.93 (0.82–1.06)
Education attainment				
No education (RC)	1.00	1.00	1.00	1.00
Primary	1.52 (1.23–1.89)***	1.56 (1.35–1.79)***	1.49 (1.23–1.80)***	1.58 (1.38–1.81)***
Secondary/tertiary	2.49 (2.03–3.05)***	2.18 (1.87–2.54)***	1.73 (1.45–2.07)***	1.62 (1.41–1.90)***
Employment status				
Not working (RC)	1.00	1.00	–	–
Currently working	0..49 (0.30–0.79)**	0.08 (0.53–0.84)**	–	–
Wealth quintile				
Lowest (RC)	1.00	1.00	1.00	1.00
Middle	1.06 (0.85–1.31)	1.42 (1.25–1.61)***	1.15 (0.95–1.39)	1.05 (0.93–1.18)
Highest	1.24 (1.01–1.52)*	1.74 (1.49–2.03)***	0.96 (0.80–1.14)	1.24 (1.08–1.43)**
Region				
North-central (RC)	1.00	1.00	1.00	1.00
North-east	1.94 (1.42–2.64)***	1.77 (1.49–2.09)***	2.37 (1.86–3.02)***	2.59 (2.21–3.03)***
North-west	1.11 (0.87–1.40)	1.26 (1.08–1.47)**	2.66 (2.17–3.27)***	2.06 (1.78–2.37)***
South-east	1.40 (1.10–1.78)**	1.42 (1.11–1.82)**	1.10 (0.92–1.31)	1.23 (1.01–1.49)*
South-south	0.95 (0.72–1.25)	0.63 (0.53–0.76)***	1.11 (0.90–1.37)	0.86 (0.74–1.01)
South-west	0.66 (0.54–0.81)***	0.62 (0.51–0.75)***	1.29 (1.10–1.52)**	1.39 (1.15–1.66)***

RC, reference category

^*^p < 0.05; **p < 0.01; ***p < 0.001

#### Wife justified in asking the husband to use a condom if he has STI

The results in Table 5 showed that rural women found in polygamous unions were significantly less likely to believe that it would be justified for a woman to ask the husband to use condoms if he has STI (aOR 0.81; CI 0.73–0.90). Also, the likelihood of believing that it would be justified for a woman to ask the husband to use condoms if he has STI significantly increased among urban women who made independent decisions on their healthcare (aOR 1.70; CI 1.32–2.18). Similar results were observed among urban and rural women who made independent decisions on how to spend their earnings (aOR 2.85; CI 2.29–3.56 and aOR 3.64; CI 3.17–4.17, respectively). Our results further indicated that an increase in women's level of education and household wealth quintile significantly increased the likelihood of believing that it would be justified for a woman to ask the husband to use condoms if he has STI. Surprisingly, the likelihood of believing that it would be justified for a woman to ask the husband to use condoms if he has STI significantly reduced among rural women who were employed (aOR 0.08; CI 0.53–0.84). There were significant variations relating to the belief that it would be justified for a woman to ask the husband to use condoms if he has STI among urban and rural women by region of residence. For instance, the likelihood of believing that it would be justified for a woman to ask the husband to use condoms if he has STI significantly increased among urban and rural women in the North-east and South-east regions, but reduced among women in the South-west region (aOR 0.66; CI 0.54–0.81 and aOR 0.62; CI 0.51–0.75, respectively).

#### Refusing to have sex with the husband if he had sex with other women

Our results in Table 5 further confirmed previous results in Tables 3 and 4 which showed that urban and rural women found in polygamous unions were less likely to believe that a woman is justified in refusing to have sexual intercourse with her husband if she knows that he has sexual intercourse with other women (aOR 0.62; CI 0.54–0.71 and aOR 0.73; CI 0.66–0.81, respectively). A similar result was observed among rural women who made independent decisions on their healthcare (aOR 0.52; CI 0.44–0.62) relative to those in the reference category. On the other hand, the likelihood of believing that a woman is justified in refusing to have sexual intercourse with her husband if she knows that he has sexual intercourse with other women significantly increased among rural women who made independent decisions on large household purchases (aOR 1.29; CI 1.04–1.61); and urban and rural women who made independent decisions on how to spend their earnings (aOR 1.42; CI 1.15–1.74 and aOR 2.51; CI 2.19–2.86, respectively).

The results in Table 5 further showed that an increase in women's level of education significantly increased the likelihood of believing that a woman is justified in refusing to have sexual intercourse with her husband if she knows that he has sexual intercourse with other women. A similar result was observed among rural women who were found in the highest wealth quintile households (aOR 1.24; CI 1.08–1.43) compared to those in the reference category. With respect to the region of residence, the likelihood of believing that a woman is justified in refusing to have sexual intercourse with her husband if she knows that he has sexual intercourse with other women significantly among urban and rural women living in the North-east, North-west and South-west, as well as rural women in the South-east regions.

## Discussion

The objective of this paper was to examine the roles of family structure and decision-making autonomy on women’s attitudes towards negotiating safe sexual practices in Nigeria. Our study revealed that a large proportion of urban and rural women believed that it would be justified for a woman to ask the husband to use condoms if he has STI and in refusing to have sexual intercourse with her husband if she knows that he has sexual intercourse with other women. Most of the women were found in monogamous unions, though higher in urban areas compared to rural areas. The disparity by place of residence could be attributable to the burden of raising large families, especially in urban areas. Also, most women lacked decision-making autonomy on their healthcare and large household purchases, while women decision-making autonomy was strongly displayed on how to spend their earnings.

It emerged from our study that differences in family structure brought about variations in having attitudes towards negotiating safe sexual practices among women. For instance, coming from polygamous unions was perceived not to be a protective factor for negotiating safe sexual practices compared to their counterparts in monogamous unions in most marriages. The plausible explanation for this observation could be attributed to the existence of competition among wives to win the husband’s affection and attention in polygamous unions as a result of the presence of other women in the family. As a result, most women might be constrained not to oppose their partners’ positions on issues relating to sexual practices [6, 10]. The findings of this study have some policy implications because the presence of a younger wife is perceived to be a threat to older wives in polygamous unions, hence exposing and compelling them to the risk of engaging in unsafe sexual practices with partners [7, 10]. Attributably, the fear of being neglected by their partners and losing them to other women encourages negative attitudes towards negotiating safe sexual practices among most women in marriages [11]. This further has implications for the high fertility rate since having many children is reportedly a powerful strategy for a woman to get more leverage over her co-wives and husbands, even against her wish to have more children.

Our study revealed that urban women who enjoyed decision-making autonomy on their healthcare were significantly more likely to believe that it would be justified for a woman to ask the husband to use condoms if he has STI, while rural women who made independent decisions on their healthcare were significantly less likely to believe that it would be justified in refusing to have sexual intercourse with her husband if she knows that he has sexual intercourse with other women. This could be attributed to the variations in educational attainment and employment of urban and rural women. Education provides women with employment opportunity and helps to provide most women with the awareness that helps them to exhibit safe sexual behaviours in most sexual relationships. The empowerment of women is an important tool enabling them to have desirable access to sexual and reproductive and health care services, including contraceptive use aimed at improving mother and child health outcomes. The results plausibly indicate that giving women the decision-making autonomy, especially rural women to decide on their healthcare could discourage them from making healthy and proper decisions on sexual matters in most marriages. This could be attributed to the culture of male dominance and women subordination to men predominantly in rural areas which encourages African women in marital or sexual unions to abide by their partners’ decisions to ensure stability in relationships [10, 21]. In addition, the demands for safe sexual practices by women who enjoyed decision-making autonomy on their healthcare might be portrayed as a sign of mistrust or infidelity in marriages [12].

Concerning socio-economic and demographic variables, our study showed significant direct relationships between women’s educational attainment and attitudes towards negotiating safe sexual practices among women in urban and rural areas. This corroborates previous studies in the observation that education influences people’s ability to adopt attitudes and behaviours that enable them to revoke cultural norms and values that promote inequality in marriage [22]. For employment status, the fact that the likelihood of believing that it would be justified for a woman to ask the husband to use condoms if he has STI significantly reduced among urban and rural women who were employed has some policy implications [23]. This could explain the negative influence of women’s exposure and swap of ideas in the workplaces on issues relating to sexual and reproductive health. Our study further showed that an increase in wealth quintile households increases the likelihood of urban and rural women having positive attitudes towards negotiating safe sexual practices in marriage, especially asking the husband to use condoms if he has STI. Consequently, earning more income by women provides them with some leverage in initiating safe sexual practices. Hence, the need to encourage and introduce more women education and empowerment programmes geared towards promoting safe sexual practices in marriage. In addition, the results on some substantial regional variations in negotiating safe sexual practices among women in Nigeria could be attributed to region-level factors including differences in cultural practices, social norms and values relating to sexual relationships in marriage across the regions. This calls for urgent region-based programmes and policy attention aimed at ensuring the achievement of SDGs 3 target of ending STIs including HIV, reducing maternal mortality ratio and burden of babies born with infections from STIs in Nigeria.

### Limitations

This study has some limitations which include the use of DHS data that constrained inference of cause-effect relationship given that the data are cross-sectional. The family structure and three women’s decision-making measures associated with attitudes towards negotiating safe sexual practices are relatively temporary. As a result, there is the likelihood of reporting bias/discordance regarding the type of family structure and the level of decision-making autonomy as the participants/respondents were women. The influence of family structure and decision-making autonomy on attitudes towards negotiating safe sexual practices might be under/over-estimated when only women's reports are considered independently. Also, the survey lacked information on behavioural and cultural factors that might have influenced the estimation of the influence of family structure and decision-making autonomy on attitudes towards negotiating safe sexual practices. Despite these limitations, the findings are important for more strategic policies and programmes, especially for disadvantaged women concerning the family structure and decision-making participation influencing the negotiation for safe sexual practices in Nigeria.

## Conclusions

This study showed that family structure decision-making autonomy influence women’s attitudes towards negotiating safe sexual practices in marriages. For instance, women coming from polygamous unions were found not to be a protective factor for negotiating safe sexual practices in most marriages plausibly because of the existence of competition among wives to win the husband’s affection and attention. The variations on the attitudes towards negotiating safe sexual practices among urban and rural women, explains the disparity in educational attainment and employment opportunities, as well as the reflection of how women’s opportunity to make decisions that affect their lives influence a range of sexual practices and relationship power. Also, women who enjoy decision-making autonomy on issues relating to their healthcare, especially rural women might be constrained to compromise their positions in sexual relationships with partners in most marriages because of the culture of male dominance and women subordination to men that are predominant in rural areas.

Given the protective factors of some women’s socio-economic and demographic characteristics to having positive attitudes towards negotiating safe sexual practices, there is the need for the creation of supportive environments in which such attitudes are transformed into behaviour with consideration of region-level factors including cultural and social norms differences which have grave implications for vulnerability to STI in marriages. Also, there should be more quantitative and qualitative studies based on the disaggregation of the data between the geopolitical zones, to explore contextual and socio-cultural factors influencing women’s attitudes towards negotiating safe sexual practices in a patriarchal society like Nigeria where most women lack decision-making autonomy on their sexual and reproductive health.

### Policy implications

The findings of this study have several policy implications for STIs’ prevention in the future among ever-married or cohabiting women in Nigeria. Both government and non-government organisations should look beyond the existing health care programmes to intensify efforts of spreading the reach of pooled resources and income-generating projects that are geared towards empowering women to gain a higher ground/voice in negotiating safe sexual practices in marital unions. No doubt, adopting the implications of these findings are essential for futuristic strategies towards STIs’ prevention by empowering women, especially the disadvantaged to air their views in marriages as STIs disproportionately affect women in Nigeria. This would enable policy-makers to develop more strategies towards achieving the SDGs’ targets of preventing deaths of newborns, ending STIs, and creating gender parity in Nigeria.

## Data Availability

The NDHS 2018 individual recode dataset was used for this study and is freely available from the DHS Program archive at https://www.dhsprogram.com/data/dataset.

## Abbreviations

AIDS: Acquired immunodeficiency virus
aOR: Adjusted odds ratio
CI: Confidence intervals
DHS: Demographic and Health Survey
HIV: Human immunodeficiency virus
NDHS: Nigeria Demographic and Health Survey
OR: Unadjusted odds ratio
RC: Reference category
SDGs: Sustainable development goals
STIs: Sexually transmitted infections
UNAIDS: The Joint United Nations Programme on HIV and AIDS
WHO: World Health Organisation
